# In silico prediction of the neutralization range of human anti-HIV monoclonal antibodies

**DOI:** 10.1186/1742-4690-9-S2-P332

**Published:** 2012-09-13

**Authors:** E Shmelkov, C Krachmarov, A Grigoryan, A Agarwal, A Statnikov, T Cardozo

**Affiliations:** 1 New York University School of Medicine, New York, NY, USA; 2 University of Medicine and Dentistry of New Jersey, Newark, NJ, USA; 3 NYU Center for Health Informatics and Bioinformatics, New York, NY, USA

## Background

Antigenic variation is a primary obstacle to HIV-1 vaccine development since antibodies (Ab) directed against the viral envelope have widely variable and poorly predictable cross-strain reactivity. The breadth of cross-strain reactivity is usually estimated by in vitro neutralization of a broad panel of HIV-1 viral strains by a query antibody. However, this approach is cumbersome and cannot be scaled up to assess the more than 60,000 circulating HIV-1 viruses.

## Methods

To address this issue, we used in silico docking of a flexible peptide, representing the epitope-containing part of a viral gp120, to a static crystallographic conformation of an antigen-combining site of an Ab. This procedure was applied to predict whether neutralization would occur between the pair. To train the prototype method we used a panel of 59 V3 sequence diverse pseudoviruses (psVs) controlled for masking effects. All psVs had an associated experimentally derived IC50 value for neutralization by anti-V3 monoclonal Abs 2219 and 447-52D.

## Results

We optimized the method for each of the two Abs by determining an optimal docking model (optimal boundaries of a docking peptide and an optimal Ab crystallographic conformation) giving the largest area under the prediction ROC curve (AUC) on the training set of 59 psVs. The prediction accuracy for the optimized method was then estimated: the AUC was equal to 0.96 (95% CI (0.91; 1)) for 2219, and to 0.88 (95% CI (0.79; 0.97)) for 447-52D.

## Conclusion

The method accurately predicts the neutralization of any HIV-1 strain by mAbs 2219 or 447-52D based solely on neutralization assay independent energetics and 3D structural parameters. The neutralization range of these anti-V3 mAbs can therefore be precisely determined in silico. Furthermore, given the fact that mAbs 2219 and 447-52D have completely different binding modes, we anticipate that our approach is extensible to other antibody-viral complexes with known structure.

